# Prognostic and Immunological Significance of CXCR2 in Ovarian Cancer: A Promising Target for Survival Outcome and Immunotherapeutic Response Assessment

**DOI:** 10.1155/2021/5350232

**Published:** 2021-11-19

**Authors:** Haizhou Ji, Mi Ren, Tongyu Liu, Yang Sun

**Affiliations:** ^1^Department of Gynecologic Oncology, Fujian Medical University Cancer Hospital, Fujian Cancer Hospital, Fuzhou, 350014 Fujian, China; ^2^Department of Oncological Nursing, Fujian Medical University Cancer Hospital, Fujian Cancer Hospital, Fuzhou, 350014 Fujian, China

## Abstract

**Objective:**

Uncovering genetic and immunologic tumor features is critical to gain insights into the mechanisms of immunotherapeutic response. Herein, this study observed the functions of CXCR2 in prognosis and immunology of ovarian cancer.

**Methods:**

Expression, prognostic significance, and genetic mutations of CXCR2 were analyzed in diverse cancer types based on TCGA and GTEx datasets. Associations of CXCR2 expression with immune checkpoints, neoantigens, tumor mutational burden (TMB), and microsatellite instability (MSI) were evaluated across pancancer. CXCR2-relevant genes were identified, and their biological functions were investigated in ovarian cancer. Through three algorithms (TIMER, quanTIseq, and xCell), we assessed the relationships of CXCR2 with immune cell infiltration in ovarian cancer. GSEA was adopted for inferring KEGG and hallmark pathways involved in CXCR2.

**Results:**

CXCR2 presented abnormal expression in tumors than paired normal tissues across pancancer. Higher expression of CXCR2 was found in ovarian cancer. Moreover, its expression was in relation to overall survival and progression including ovarian cancer. Prominent associations of CXCR2 with immune checkpoints, neoantigens, TMB, and MSI were observed in human cancers. Somatic mutations of CXCR2 frequently occurred across pancancer. Amplification was the main mutational type of CXCR2 in ovarian cancer. CXCR2-relevant genes were markedly enriched in immunity activation and carcinogenic pathways in ovarian cancer. Moreover, it participated in modulating immune cell infiltration in the tumor microenvironment of ovarian cancer such as macrophage and immune response was prominently modulated by CXCR2.

**Conclusion:**

Collectively, CXCR2 acts as a promising prognostic and immunological biomarker as well as a novel immunotherapeutic target of ovarian cancer.

## 1. Introduction

Ovarian cancer represents the major cause of deaths of gynecological malignancies [1, 2]. Epithelial ovarian cancer is the most common form [3]. The five-year survival rate is <35% globally [3]. 70% of affected patients have advanced-stage disease [4]. The present first-line standards of care include debulking surgery plus platinum–taxane maintenance chemotherapeutic strategy [5]. Following the first-line treatment, cancer may relapse among 60–70% of patients with first-rank debulking as well as 80–85% of patients with suboptimal debulking [6]. The high mortality of ovarian cancer patients can be attributed to chemotherapy resistance, extensive intraperitoneal metastasis, and other factors [7]. Awful mortality may be attributed chemotherapeutic resistance, extensive intraperitoneal metastases, etc. [8]. Tumor microenvironment exerts a critical role in the progression and clinical outcomes of ovarian cancer [9]. Emerging immunotherapeutic strategies enhance the antitumor immune response by diverse methods such as immunostimulatory cytokine and tumor antigen vaccine as well as monoclonal antibody [10]. Though immunotherapy-relevant agents like olaparib may ameliorate ovarian cancer progression, there is no prominent breakthrough for its effective therapy [11]. The presence and absence of tumor-infiltrating lymphocytes are separately judged as “hot” tumor and “cold” tumor [12]. Therefore, because of high infiltration levels of tumor-infiltrating lymphocytes, “hot” tumor can respond to immunosuppressive checkpoint inhibitors. Nevertheless, despite the relatively increased tumor mutation burden (TMB) of ovarian cancer, it is still a “cold” tumor [13]. Thus, it is of importance to explore how to activate the immune system in “cold” tumor such as T cell and tumor-associated macrophage.

Chemokine receptor (CXCR) family (including CXCR1-7) is a type of G-protein-coupled receptors, abundant in 7 transmembrane motifs containing hydrophobic amino acids [14]. Among them, CXCR2 was originally thought to be a G protein-coupled transmembrane chemokine receptor expressed on neutrophil [15]. It possesses the functions in various leukocytes such as neutrophil, eosinophil, and monocyte as well as macrophage, which is related to immune response [16]. Targeting CXCR2 in myeloid-derived suppressor cells may improve antitumor immune response [17]. Emerging evidence suggests that CXCR2 is involved in the recruitment of immune cells as well as promotes angiogenesis, tumor growth, and metastases [18]. It exhibits high affinity for IL-8 and Gro-1 but low affinity for Gro-2, Gro-3, and epithelial neutrophil-activating peptide-78 [18]. Moreover, high expression of CXCR2 contributes to carcinogenesis in diverse cancer types, especially ovarian cancer [19]. Also, CXCR2-expressing ovarian cancer is aggressive with undesirable clinical outcomes [20]. CXCR2 is crucial for the acquisition of cisplatin chemoresistance of ovarian cancer cells [21]. Despite this, the functions of CXCR2 in prognosis and immunology in ovarian cancer remain ambiguous. In this study, we aimed to evaluate the prognostic and immunological significance of CXCR2 in ovarian cancer.

## 2. Materials and Methods

### 2.1. Data Acquisition

This study acquired the transcriptome data, follow-up information, and genetic mutation data of pancancer samples from The Cancer Genome Atlas (TCGA) project via Genomic Data Commons (GDC) web server [22]. Meanwhile, we curated transcriptome profiles of normal specimens from Genotype-Tissue Expression (GTEx) projects [23]. Gene Expression Profiling Interactive Analysis 2 (GEPIA2) web server (http://gepia2.cancer-pku.cn/) provides an accessible resource for gene expression analysis in tumor and normal specimens from TCGA and GTEx projects. CXCR2 expression was compared between tumor and normal specimens with the Wilcoxon test. “Survival” module of GEPIA was applied for assessing the correlations of CXCR2 expression with overall survival (OS) of diverse cancer types. CXCR2 expression was analyzed across distinct cancer pathological stages. Univariate cox regression analyses were presented for investigating the associations of CXCR2 expression with OS and disease-specific survival (DSS) for diverse cancer types in TCGA cohort.

### 2.2. Analysis of Associations between CXCR2 Expression and Immune Checkpoints, Neoantigens, TMB, and Microsatellite Instability (MSI)

The known immune checkpoints were curated from previous research [24]. TMB was calculated as the number of somatic, coding, base substitutions, and insert-deletion alterations per megabase of the genome detected utilizing nonsynonymous and code-shifting indels with the detection limit of 5% [25]. The formula of TMB was as follows: TMB = Sn × 1,000,000/*n*, where Sn represented the absolute number of somatic alterations while *n* represented the number of exon base coverage depth ≥ 100×. The number of neoantigens [26] as well as MSI [27] was separately counted across pancancer. Through the Spearman correlation test, we assessed the associations of CXCR2 expression with immune checkpoints, neoantigens, TMB, and MSI in diverse cancer types.

### 2.3. Somatic Mutation Analysis

Somatic mutations were visualized across ovarian cancer specimens from TCGA dataset utilizing Maftools package [28]. Through cBio Cancer Genomics Portal (cBioPortal) platform (http://cbioportal.org/) [29], alteration frequency of CXCR2 was analyzed in diverse cancer types. Genomic mutations of CXCR2 contained copy number amplification, deep deletion, and missense mutation.

### 2.4. Differential Expression Analysis

In line with the median value of CXCR2 expression, ovarian cancer specimens in TCGA dataset were classified into high- and low-expression groups. Limma package (version 3.40.2) was adopted for differential expression analysis between two groups [30]. With ∣log2fold change | >1 and false discovery rate (FDR) < 0.05, CXCR2-relevant genes were identified in ovarian cancer.

### 2.5. Function Enrichment Analysis

Biological processes of Gene Ontology (GO) and Kyoto Encyclopedia of Genes and Genomes (KEGG) pathways enriched by CXCR2-relevant genes were analyzed with clusterProfiler package [31]. Terms with FDR < 0.05 were significantly enriched.

### 2.6. Analysis of Immune Cell Infiltration

Three algorithms including Tumor Immune Estimation Resource (TIMER; http://cistrome.shinyapps.io/timer) [32], quanTIseq (http://icbi.at/quantiseq) [33], and xCell (http://xCell.ucsf.edu/) [34] were employed for inferring the infiltration levels of immune cells in ovarian cancer from TCGA dataset. The Spearman correlation test was utilized to evaluate the relationships of CXCR2 expression with immune cell infiltrations.

### 2.7. Gene Set Enrichment Analysis (GSEA)

For exploring the biological signaling pathways involved in CXCR2, GSEA software (version 4.0.3) [35] was adopted carried out between high- and low-expression groups with the median value of CXCR2 expression as the cutoff value. The first three or four terms of KEGG and hallmark were visualized. The gene sets of KEGG and hallmark pathways were curated from the Molecular Signature Database (MsigDB; http://www.broadinstitute.org/msigdb) [36]. KEGG or hallmark pathways with ∣nominal enrichment score (NES) | >1.7 and nominal *p* < 0.05 were considered to have significant enrichment.

### 2.8. Statistical Analysis

All statistics were presented with R software (version 4.0.3; https://www.R-project.org) and its packages. Comparisons between groups were conducted with Student's *t*-test, the Wilcoxon test, or one-way variance analyses. The Spearman or Pearson correlation test was utilized for evaluating correlations between variables. A *p* value < 0.05 was set as statistically significant.

## 3. Results

### 3.1. Expression Patterns of CXCR2 across Pancancer

**Figure 1:**
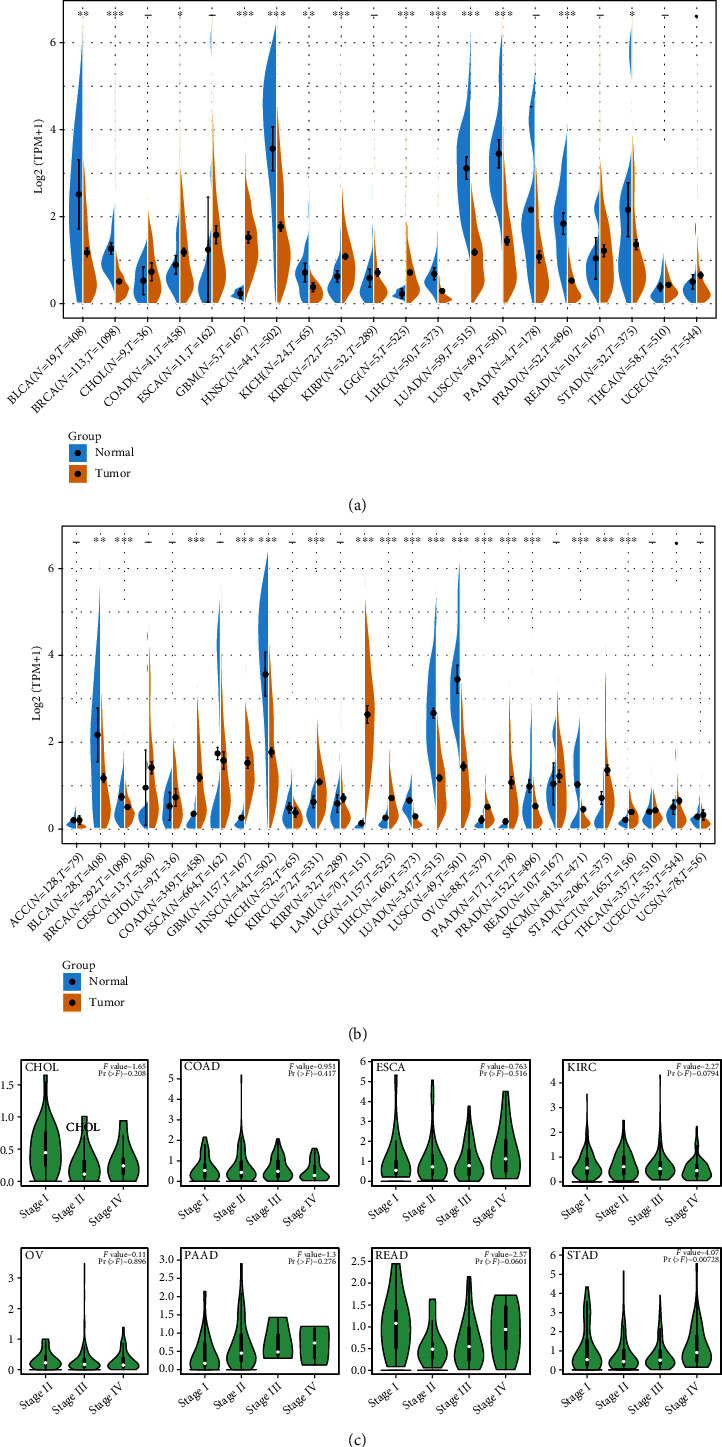
Expression patterns of CXCR2 across pancancer. (a) Expression levels of CXCR2 in tumor and normal tissues in TCGA dataset. (b) Expression levels of CXCR2 in tumor and normal tissues in TCGA and GTEx datasets. Yellow fusiformis represented tumor samples while blue fusiformis represented normal samples. The *X*-axis meant number of tumor and normal specimens. The *Y*-axis meant log2(transcript per million (TPM) + 1). ^∗^*p* < 0.05; ^∗^*p* < 0.01; ^∗∗∗^*p* < 0.001. (c) Expression levels of CXCR2 in different pathological stages across pancancer.

Herein, this study evaluated the expression of CXCR2 in diverse tumor tissues and matched normal tissues. We collected data from TCGA and GTEx datasets. In TCGA dataset, we noticed high expression of CXCR2 in COAD, GBM, KIRC, and LGG (Figure 1(a)). In contrast, CXCR2 displayed reduced expression in BLCA, BRCA, HNSC, KICH, LIHC, LUAD, PAAD, PRAD, and STAD. Due to the relatively small sample size of normal tissues in TCGA, we integrated data from TCGA and GTEx datasets. There was reduced expression of CXCR2 in BLCA, BRCA, COAD, GBM, HNSC, LIHC, LUAD, LUSC, PRAD, and SKCM (Figure 1(b)). Nevertheless, upregulation of CXCR2 expression was found in KIRC, LAML, LGG, OV, PAAD, STAD, and TGCT. Using the GEPIA2 tool, the relationships of CXCR2 expression with pathological staging were evaluated in CHOL, COAD, ESCA, KIRC, OV, PAAD, READ, and STAD (Figure 1(c)). Among them, CXCR2 displayed stage-specific expression alterations in STAD, while no clear associations were found in most cancer types.

### 3.2. Prognostic Significance of CXCR2 across Pancancer

**Figure 2:**
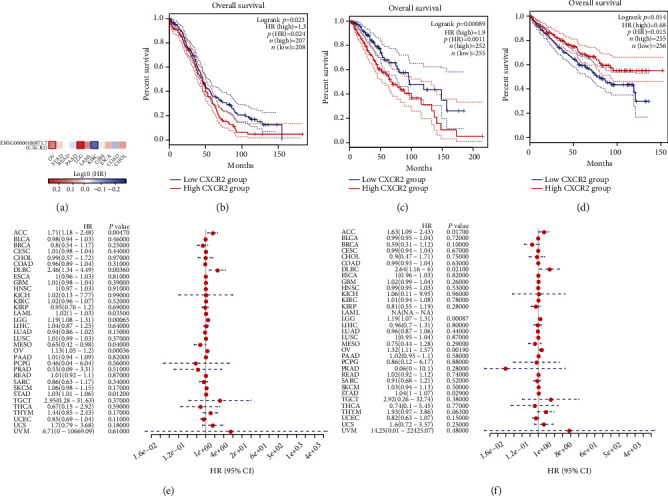
Evaluation of prognostic significance of CXCR2 across pancancer. (a) Survival map for the associations of CXCR2 with OS of diverse cancer types. Red meant HR > 1 while blue meant HR < 1. (b–d) Kaplan-Meier curves of high and low expression of CXCR2 groups for OV, LGG, and KIRC patients. (e, f) Univariate cox regression analysis showing the associations of CXCR2 with OS and DSS across diverse cancer types.

Through the GEPIA2 tool, we investigated the associations of CXCR2 with OS across diverse cancer types. The results demonstrated that CXCR2 upregulation was in relation to worse OS of OV and LGG patients (Figures 2(a)–2(c)). Oppositely, KIRC patients with high expression of CXCR2 displayed marked survival advantage in comparison to those with low expression of CXCR2 (Figure 2(d)). Furthermore, univariate cox regression models were conducted for investigating the correlations of CXCR2 with OS and DSS in each cancer type. In Figure 2(e), we noticed CXCR2 as a risk factor for OS of ACC, DLBC, LAML, LGG, OV, and STAD. In contrast, CXCR2 acted as a protective factor of MESO OS. Moreover, CXCR2 upregulation displayed worse DSS for ACC, DLBC, LGG, OV, and STAD (Figure 2(f)). Kaplan-Meier curves also demonstrated the prognostic significance of CXCR2 in OS and DSS of diverse cancer types (Supplementary figure 1A, B).

### 3.3. Analysis of Links between CXCR2 Expression and Tumor Immune Response across Pancancer

**Figure 3:**
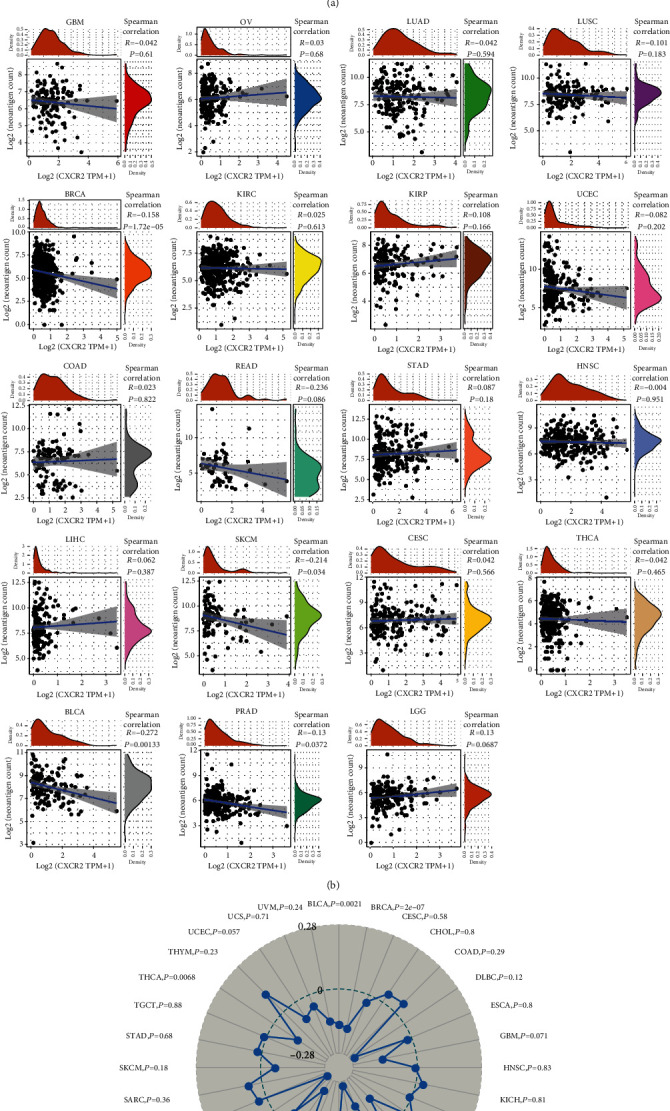
Associations of CXCR2 expression with tumor immune response across pan-cancer. (a) Correlations of CXCR2 expression with acknowledged immune checkpoint genes in diverse cancer types. The lower triangle meant coefficients calculated with Pearson's correlation test, while the upper triangle meant *p* value. ^∗^*p* < 0.05, ^∗∗^*p* < 0.01, and ^∗∗∗^*p* < 0.001. (b) Correlation analysis between CXCR2 expression and the number of immune neoantigens across pancancer. (c) Association analysis of CXCR2 expression with TMB across pancancer utilizing Spearman's correlation test. (d) Association analysis of CXCR2 expression with MSI in diverse cancer types with Spearman's correlation test.

Nowadays, several genes have been recognized as immune checkpoints in tumor immune response. We evaluated whether there is a link of CXCR2 with immune checkpoint genes. The results demonstrated markedly positive associations between CXCR2 and immune checkpoint genes such as CD86, VSIR, CD28, and CTLA4 across pancancer (Figure 3(a)). For uncovering the function of CXCR2 in the immune mechanism and immune response, this study evaluated the interactions of CXCR2 expression with neoantigens, TMB, and MSI. Neoantigens, TMB, and MSI are in relation to antitumor immunity and may predict therapeutic responses to immunotherapeutic agents. Correlation between CXCR2 expression and neoantigens was assessed in diverse cancer types. In Figure 3(b), CXCR2 exhibited prominently negative associations with the number of neoantigens in BRCA, SKCM, BLCA, and PRAD. Moreover, we noticed the negative links of CXCR2 expression with TMB in BLCA, BRCA, LIHC, LUAD, PAAD, PRAD, and THCA (Figure 3(c)). However, CXCR2 expression displayed positive correlations to TMB in LGG and OV. As depicted in Figure 3(d), there were negative interactions between CXCR2 expression and MSI in CHOL, ESCA, HNSC, KIRP, LGG, LUAD, LUSC, PAAD, PRAD, SKCM, STAD, UCEC, and UCS. The above evidence highlighted the implications of CXCR2 expression in tumor immune response.

### 3.4. Analysis of Somatic Mutation of CXCR2 in Ovarian Cancer

**Figure 4:**
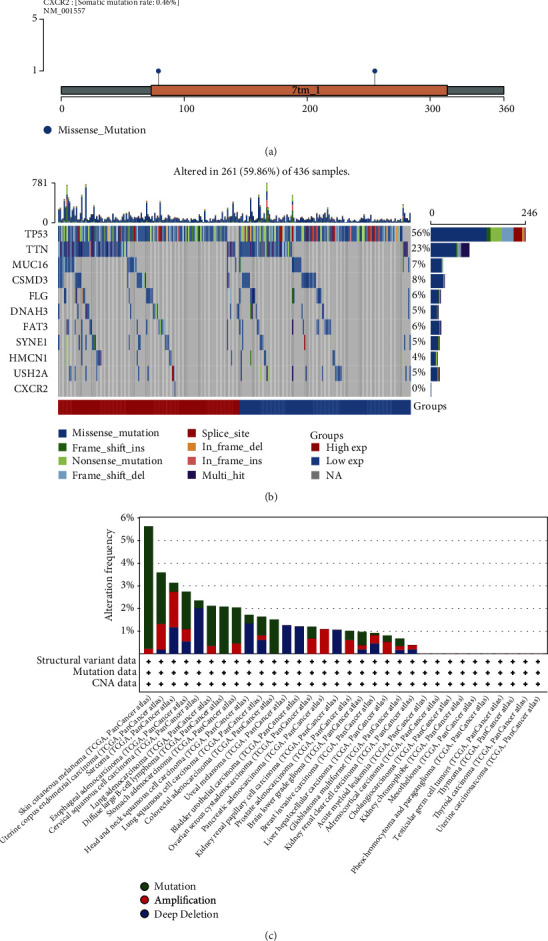
Analysis of somatic mutation of CXCR2 in ovarian cancer. (a) Somatic mutation rate of CXCR2 across ovarian cancer. (b) Landscape of genetic mutations across ovarian cancer specimens. Waterfall plots showed the mutational information of each gene in each specimen. Diverse colors at the bottom represented diverse mutational types. The barplot above the legend displayed the number of mutational burden. (c) Genetic mutation type and frequency of CXCR2 across pancancer via the cBioPortal tool. Histogram showed the alteration frequencies of CXCR2 in diverse cancer types. Green, mutation; red, amplification; and blue, deletion.

We analyzed the somatic mutation of CXCR2 in ovarian cancer. As shown in Figure 4(a), the somatic mutation rate was 0.46%. Among 436 ovarian cancer samples, genetic mutations occurred in 261 (59.86%) (Figure 4(b)). TP53 (56%), TTN (23%), CSMD3 (8%), MUC16 (7%), FLG (6%), FAT3 (6%), DNAH3 (5%), SYNE1 (5%), USH2A (5%), and HMCN1 (4%) were the most frequently mutated genes across ovarian cancer. Moreover, missense mutation was the major mutation type. However, no significant difference in genetic mutation was investigated between high and low expression of CXCR2 groups. Through cBioPortal tool, we evaluated the genetic mutation of CXCR2 across pancancer. We noticed that amplification of CXCR2 occupied the relatively high alteration frequency in ovarian cancer, which could contribute to the upregulation of CXCR2 expression (Figure 4(c)).

### 3.5. Identifying CXCR2-Relevant Genes and Their Biological Significance

**Figure 5:**
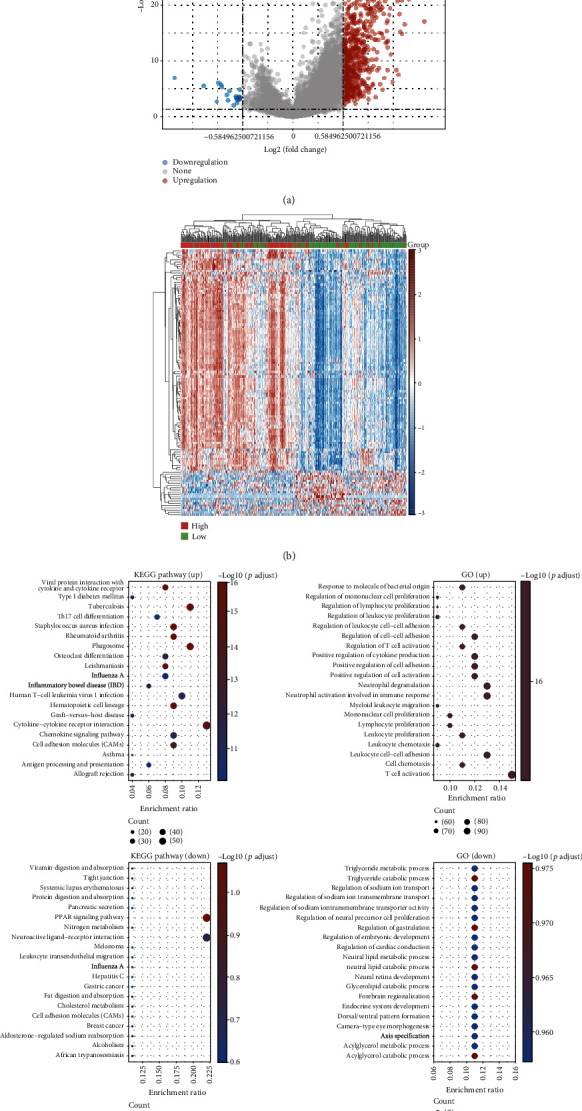
Identifying CXCR2-relevant genes in ovarian cancer and their biological significance. (a, b) Volcano plots and heat map visualized the expression patterns of CXCR2-relevant genes in high and low expression of CXCR2 groups. Red meant upregulation; blue meant downregulation; and grey meant no significant difference. (c) KEGG pathways and biological processes enriched by upregulated CXCR2-relevant genes or downregulated CXCR2-relevant genes.

To identify CXCR2-relevant genes, we separated ovarian cancer patients into high- and low-expression groups in line with the median value of CXCR2 expression. With ∣log2fold change | >1 and FDR < 0.05, we screened 734 CXCR2-relevant genes (Figures 5(a) and 5(b)). Among them, 715 genes were upregulated while 19 genes were downregulated in the high-expression group (Tables 1 and 2). Function enrichment analysis was presented for uncovering the biological significance of CXCR2-relevant genes. In Table 3 and Figure 5(c), upregulated genes were in relation to KEGG pathways of immunity and inflammatory response such as Th17 cell differentiation, cytokine-cytokine receptor interaction, chemokine signaling pathway, antigen processing and presentation, human T cell leukemia virus 1 infection, graft-versus-host disease, and allograft rejection. Meanwhile, upregulated genes were prominently enriched by immune response like regulation of mononuclear, lymphocyte, and leukocyte proliferation; leukocyte cell-cell adhesion; T cell activation; myeloid leukocyte migration; neutrophil degranulation; and neutrophil activation involved in immune response (Table 4 and Figure 5(c)). Intriguingly, downregulated genes displayed significant correlations to carcinogenic pathways such as PPAR signaling pathway, neuroactive ligand-receptor interaction, melanoma, gastric cancer, cell adhesion molecules, and breast cancer (Table 5 and Figure 5(c)). Also, we noticed that downregulated genes were markedly associated with metabolic processes like triglyceride metabolic and catabolic processes, neutral lipid metabolic and catabolic processes, glycerolipid catabolic process, and acylglycerol metabolic and catabolic processes (Table 6 and Figure 5(c)).

### 3.6. Associations of CXCR2 with Immune Cell Infiltration in Tumor Microenvironment

**Figure 6:**
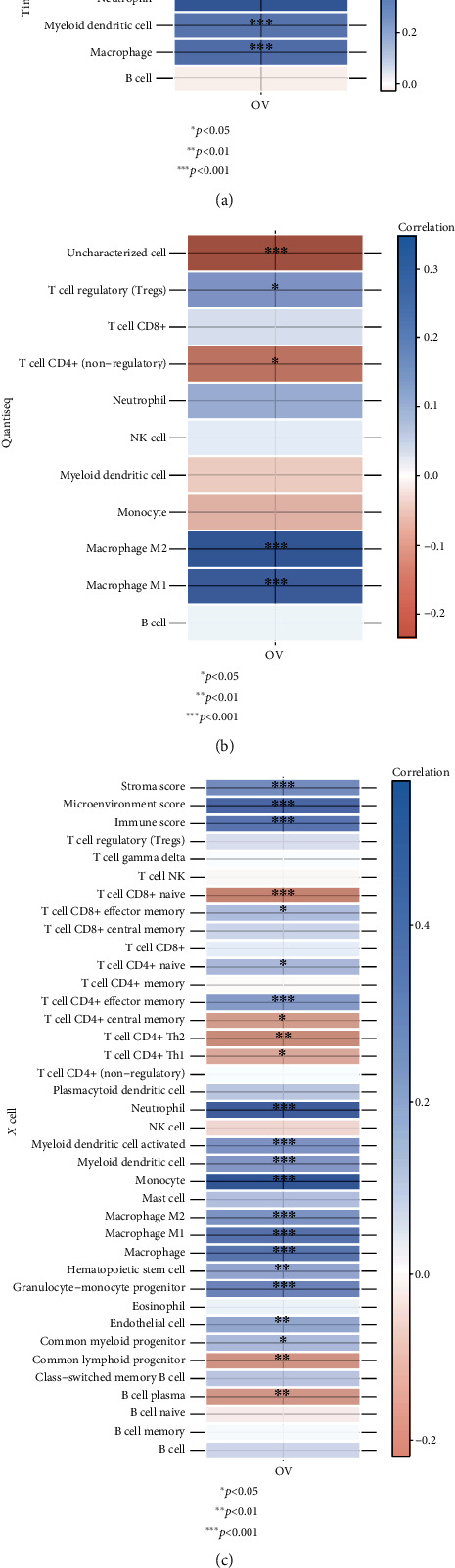
Analysis of interactions between CXCR2 and immune cell infiltration in tumor microenvironment. (a) Correlations of CXCR2 with the abundance of immune cells in ovarian cancer through TIMER algorithm. (b) Associations of CXCR2 with the abundance of immune cells across ovarian cancer tissues with quanTIseq algorithm. (c) Associations between CXCR2 and infiltration levels of immune cells in ovarian cancer utilizing xCell algorithm. Red meant positive correlation while blue meant negative correlation. ^∗^*p* < 0.05; ^∗∗^*p* < 0.01; ^∗∗∗^*p* < 0.001.

Three algorithms (TIMER, quanTIseq, and xCell) were adopted for inferring the infiltration levels of immune cells in ovarian cancer. In Figure 6(a), correlation analysis uncovered that CXCR2 was negatively associated with the abundance of CD4+ T cell, neutrophil, myeloid dendritic cell, and macrophage in ovarian cancer tissues with TIMER algorithm. Using a quanTIseq method, we noticed the negative associations of CXCR2 with T regulatory cell (Treg), M1 macrophage, and M2 macrophage (Figure 6(b)). Oppositely, there were positive correlations of CXCR2 with uncharacterized cell and CD4+ T cell. Through xCell algorithm, we investigated that CXCR2 displayed negative associations with stromal score, microenvironment score, immune score, CD8+ effector memory T cell, CD4+ naïve T cell, CD4+ effector memory T cell, neutrophil, activated myeloid dendritic cell, myeloid dendritic cell, monocyte, M1 macrophage, M2 macrophage, macrophage, hematopoietic stem cell, granulocyte-monocyte progenitor, endothelial cell, and common myeloid progenitor (Figure 6(c)). In contrast, we noticed the positive associations of CXCR2 with the abundance of CD8+ naïve T cell, CD4+ central memory T cell, CD4+ Th2 T cell, CD4+ Th1 T cell, common lymphoid progenitor, and B cell plasma across ovarian cancer. Based on three algorithms, CXCR2 expression negatively modulated macrophage infiltration in ovarian cancer.

### 3.7. Analysis of Signaling Pathways Involved in CXCR2

**Figure 7:**
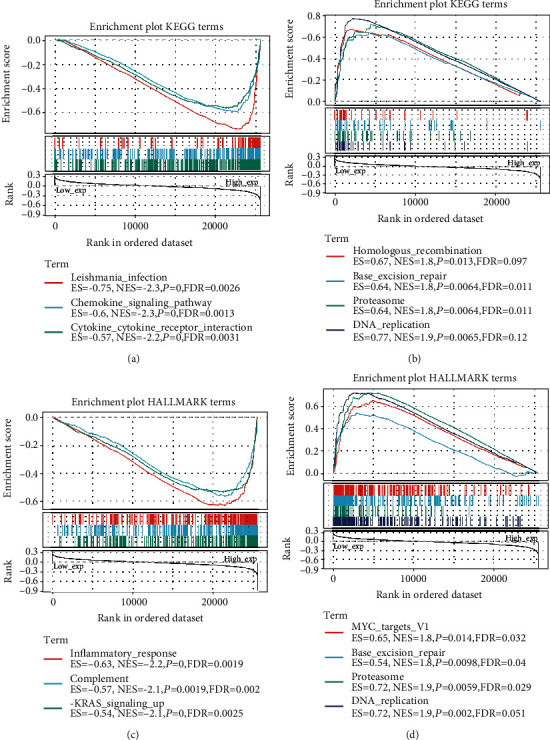
GSEA of CXCR2 linked with KEGG and hallmark pathways. (a) GSEA results of CXCR2 ranked in the first three for its negative associations with KEGG pathways. (b) GSEA results of CXCR2 ranked in the first four for its positive associations with KEGG pathways. (c) GSEA results of CXCR2 ranked in the first three for its negative associations with hallmark pathways. (d) GSEA results of CXCR2 ranked in the first four or its positive associations with hallmark pathways. ES: enrichment score; NES: nominal enrichment score; FDR: false discovery rate.

For observing the function of CXCR2 expression on tumor progression, this study separated ovarian cancer specimens into high- and low-expression groups in line with CXCR2 expression. Afterwards, we evaluated the enrichment of KEGG and hallmark pathways in high- and low-expression groups via GSEA. Our data suggested that CXCR2 exhibited negative correlations to leishmania infection, chemokine signaling pathway, and cytokine-cytokine receptor interaction KEGG pathways (Figure 7(a)). Meanwhile, there were positive relationships of CXCR2 with homologous recombination, base excision repair, proteasome, and DNA replication (Figure 7(b)). As depicted in Figure 7(c), hallmark pathways of inflammatory response, complement, and KRAS signaling up displayed negative interactions with CXCR2. In contrast, CXCR2 was in positive relation to MYC targets v1, base excision repair, proteasome, and DNA replication (Figure 7(d)).

## 4. Discussion

Based on TCGA and GTEx datasets, we observed the abnormal expression of CXCR2 in tumors and paired normal tissues across pancancer. Survival analysis uncovered the prominent prognostic significance of CXCR2 in diverse cancer types. Especially, CXCR2 expression presented marked upregulation in ovarian cancer as well as its upregulation contributed to more undesirable survival outcomes. Hence, CXCR2 might act as a promising prognostic predictor of ovarian cancer. The response of ovarian cancer to immunotherapeutic agents remains limited. Although immunotherapy may produce a long-lasting response in a few patients, most of the patients do not respond to this therapy, covering those with PD-L1-expressed tumors [37]. Nevertheless, evaluating the sensitivity or resistance to target therapeutic populations according to stratification by cancer markers including TMB, PD-L1, tumor-infiltrating lymphocytes, and neoantigens can enhance the predictive efficacy of immunotherapeutic response [38]. Our pancancer analysis demonstrated the close interactions of CXCR2 with immune checkpoints, neoantigen, TMB, and MSI, indicating that CXCR2 could participate in modulating immune response. Our genetic mutation analysis uncovered that there occurred widespread mutations of CXCR2 across pancancer. Amplification was the major mutational type of CXCR2 in ovarian cancer. This indicated that CXCR2 amplification contributed to its overexpression in ovarian cancer.

Under the cutoffs of ∣log2fold change | >1 and FDR < 0.05, we identified 734 CXCR2-relevant genes. We noticed that CXCR2-relevant genes were markedly enriched in immunity activation such as Th17 cell differentiation, cytokine-cytokine receptor interaction, chemokine signaling pathway, and antigen processing and presentation as well as carcinogenic pathways such as PPAR signaling pathway. For instance, PARP inhibitor has emerged as a therapeutic agent against ovarian cancer according to the DNA repair vulnerability in ovarian cancer cells, which prevents the repair of DNA single-strand break as well as has generated double-strand break that is unable to be precisely repaired in tumor cells [39].

Based on three algorithms (TIMER, quanTIseq, and xCell), we noticed the prominent interaction between CXCR2 and macrophage in ovarian cancer tissues. Macrophage constitutes a key component of the tumor microenvironment [13]. Tumor-associated macrophage is macrophage produced by the infiltrations of peripheral blood mononuclear cells into solid tumor tissues, occupying a large part of tumor stromal cells [13]. Because of the increased plasticity and heterogeneity of macrophage, it has distinct biological functions in diverse tumor microenvironment, including two major phenotypes: M1 and M2 macrophages [40]. Tumor-associated macrophage is abundant in the ovarian cancer microenvironment and affects patients' survival outcomes [40]. The relationship of CXCR2 with macrophage has been reported in several cancer types. For instance, macrophage reeducation by CXCR2 inhibitors may drive senescence as well as suppress tumor progression in advanced prostate cancer [41]. CXCR2-dominated interplays between cancer cells and macrophages drive gastric cancer metastases [42].

CXCR2 was mainly involved in modulating chemokine signaling pathway, cytokine-cytokine receptor interaction, inflammatory response, and complement as well as DNA damage repair. CXCR2 produced by cancer cells induce neutrophil extracellular traps, which interferes with immune cytotoxicity [43]. CXCR2-modified CAR-T cells enhance trafficking capacity, which improves therapeutic response in hepatocellular carcinoma [44]. Blockage of CXCR2 may enhance the sensitivity and effectiveness of immunotherapy and suppress tumor progression [18]. Combining previous evidence, CXCR2 may exert a critical role in modulating immune response. Nevertheless, there are several limitations in our study. The regulatory functions of CXCR2 in ovarian carcinogenesis and tumor immunity will be investigated in in vitro and in vivo experiments. Moreover, prognostic significance of CXCR2 expression should be verified in a larger ovarian cancer cohort.

## 5. Conclusion

Collectively, our integrative analysis of CXCR2 uncovered the prominent associations of CXCR2 expression with survival outcomes, immune cell infiltration, and immune response in ovarian cancer, which could contribute to explain the function of CXCR2 in carcinogenesis and immunotherapeutic response from various perspectives.

## Figures and Tables

**Table 1:** The first 20 upregulated CXCR2-relevant genes ranked by |log2fold change|.

Genes	log2fold change	Average expression	*t*	*p* value	FDR	*B*
CYBB	1.626883	4.866193	13.2859	2.82*E*-33	3.13*E*-30	64.80304
CSF1R	1.580807	5.08034	13.53358	2.96*E*-34	4.76*E*-31	67.02763
ALOX5AP	1.572744	5.250381	11.77966	1.82*E*-27	4.82*E*-25	51.62072
LYZ	1.530011	6.245003	9.038626	8.60*E*-18	6.04*E*-16	29.71133
SLCO2B1	1.489949	3.454025	14.24883	4.06*E*-37	2.39*E*-33	73.52581
MPEG1	1.475656	3.816864	13.79154	2.78*E*-35	5.46*E*-32	69.35911
VSIG4	1.457472	5.213007	11.87514	7.96*E*-28	2.39*E*-25	52.43645
FPR3	1.439832	4.19429	11.74373	2.49*E*-27	6.48*E*-25	51.31448
LAPTM5	1.439653	7.33844	12.34081	1.35*E*-29	5.82*E*-27	56.45608
FCGR3A	1.435916	5.415789	11.59154	9.24*E*-27	2.07*E*-24	50.02226
CX3CR1	1.428444	3.051211	11.38818	5.27*E*-26	1.01*E*-23	48.30796
C3AR1	1.406321	4.279677	12.81774	1.93*E*-31	1.27*E*-28	60.6387
ITGB2	1.391398	4.898533	11.96583	3.62*E*-28	1.23*E*-25	53.21399
CD163	1.390154	4.016596	11.86577	8.64*E*-28	2.55*E*-25	52.35628
GPR34	1.38616	3.462056	13.89462	1.08*E*-35	2.91*E*-32	70.29467
FPR1	1.372222	2.86155	12.72349	4.49*E*-31	2.57*E*-28	59.80717
F13A1	1.352817	3.950676	10.18952	1.10*E*-21	1.22*E*-19	38.51989
SIGLEC1	1.343363	3.306899	11.83175	1.16*E*-27	3.17*E*-25	52.06539
FGL2	1.311918	3.734005	11.99986	2.69*E*-28	9.33*E*-26	53.50643
MNDA	1.30756	3.392344	13.16289	8.62*E*-33	8.98*E*-30	63.70358

**Table 2:** The downregulated CXCR2-relevant genes ranked by |log2fold change|.

Genes	log2fold change	Average expression	*t*	*p* value	FDR	*B*
PCP4	-1.372	4.022248	-5.43074	1.01*E*-07	1.15*E*-06	7.096412
APOA1	-1.03739	6.955593	-4.73466	3.12*E*-06	2.29*E*-05	3.80636
CLDN6	-0.88582	6.125029	-3.11872	0.001957	0.006165	-2.2451
FXYD4	-0.86449	1.906169	-5.00148	8.76*E*-07	7.46*E*-06	5.021079
FGF17	-0.83534	1.148663	-4.85379	1.78*E*-06	1.40*E*-05	4.341469
SMIM24	-0.82416	2.673542	-4.64089	4.80*E*-06	3.34*E*-05	3.393485
SIX3	-0.76623	1.360529	-3.86787	0.000129	0.000589	0.274769
MAL	-0.75402	6.521845	-3.25231	0.001249	0.004188	-1.833
FXYD7	-0.73799	2.355221	-4.24556	2.75*E*-05	0.000153	1.734303
LHX1	-0.68835	2.827502	-2.62669	0.008976	0.02269	-3.62052
PDCL2	-0.66398	1.539734	-3.70687	0.000241	0.001012	-0.3093
NUPR2	-0.65946	4.707309	-3.53281	0.000462	0.001776	-0.91478
SAMD11	-0.64936	3.063171	-3.35658	0.00087	0.003063	-1.5
FABP6	-0.64904	2.858451	-2.96587	0.003212	0.009419	-2.69647
NPW	-0.63225	5.019634	-3.64286	0.000308	0.001243	-0.53513
CA9	-0.62153	4.176407	-3.36072	0.000857	0.003025	-1.48657
H3C11	-0.6192	2.105437	-4.39824	1.42*E*-05	8.64*E*-05	2.359317
PRSS1	-0.61512	3.467221	-3.50754	0.000507	0.001923	-1.00041
DEFB126	-0.61494	1.807047	-3.64746	0.000302	0.001224	-0.51901

**Table 3:** The information of the first 20 KEGG pathways enriched by upregulated CXCR2-relevant genes.

Description	GeneRatio	BgRatio	*p* value	FDR	Size
Staphylococcus aureus infection	39/411	96/8009	7.16*E*-26	1.90*E*-23	39
Hematopoietic cell lineage	38/411	99/8009	3.70*E*-24	4.90*E*-22	38
Phagosome	44/411	152/8009	4.61*E*-22	3.62*E*-20	44
Rheumatoid arthritis	35/411	93/8009	5.46*E*-22	3.62*E*-20	35
Leishmaniasis	31/411	77/8009	1.26*E*-20	6.68*E*-19	31
Tuberculosis	45/411	180/8009	1.02*E*-19	4.52*E*-18	45
Viral protein interaction with cytokine and cytokine receptor	32/411	100/8009	9.47*E*-18	3.58*E*-16	32
Cytokine-cytokine receptor interaction	54/411	294/8009	5.80*E*-17	1.92*E*-15	54
Cell adhesion molecules (CAMs)	36/411	147/8009	1.17*E*-15	3.46*E*-14	36
Osteoclast differentiation	32/411	128/8009	2.59*E*-14	6.87*E*-13	32
Inflammatory bowel disease (IBD)	23/411	65/8009	3.66*E*-14	8.81*E*-13	23
Allograft rejection	18/411	38/8009	5.50*E*-14	1.22*E*-12	18
Chemokine signaling pathway	38/411	189/8009	1.71*E*-13	3.49*E*-12	38
Human T cell leukemia virus 1 infection	41/411	219/8009	2.24*E*-13	4.24*E*-12	41
Graft-versus-host disease	18/411	41/8009	2.87*E*-13	5.07*E*-12	18
Type I diabetes mellitus	18/411	43/8009	7.86*E*-13	1.30*E*-11	18
Th17 cell differentiation	27/411	107/8009	2.29*E*-12	3.57*E*-11	27
Antigen processing and presentation	23/411	78/8009	2.99*E*-12	4.40*E*-11	23
Influenza A	34/411	170/8009	4.03*E*-12	5.63*E*-11	34
Asthma	15/411	31/8009	4.95*E*-12	6.57*E*-11	15

**Table 4:** The information of the first 20 biological processes enriched by upregulated CXCR2-relevant genes.

Description	GeneRatio	BgRatio	*p* value	FDR	Size
T cell activation	98/670	483/18866	1.24*E*-46	4.26*E*-43	98
Leukocyte cell-cell adhesion	86/670	364/18866	1.85*E*-46	4.26*E*-43	86
Leukocyte proliferation	74/670	313/18866	5.29*E*-40	8.13*E*-37	74
Neutrophil activation involved in immune response	89/670	490/18866	2.89*E*-38	3.32*E*-35	89
Leukocyte chemotaxis	63/670	232/18866	5.31*E*-38	4.89*E*-35	63
Neutrophil degranulation	88/670	487/18866	1.19*E*-37	9.12*E*-35	88
Cell chemotaxis	71/670	311/18866	2.56*E*-37	1.68*E*-34	71
Regulation of leukocyte proliferation	63/670	240/18866	4.88*E*-37	2.81*E*-34	63
Mononuclear cell proliferation	68/670	286/18866	5.87*E*-37	3.00*E*-34	68
Regulation of leukocyte cell-cell adhesion	72/670	329/18866	1.55*E*-36	7.13*E*-34	72
Positive regulation of cytokine production	83/670	447/18866	2.02*E*-36	8.45*E*-34	83
Lymphocyte proliferation	67/670	283/18866	2.69*E*-36	1.03*E*-33	67
Positive regulation of cell adhesion	81/670	428/18866	3.58*E*-36	1.27*E*-33	81
Myeloid leukocyte migration	60/670	222/18866	4.28*E*-36	1.41*E*-33	60
Response to molecule of bacterial origin	74/670	356/18866	6.20*E*-36	1.90*E*-33	74
Regulation of T cell activation	71/670	332/18866	2.43*E*-35	7.00*E*-33	71
Regulation of mononuclear cell proliferation	59/670	221/18866	3.54*E*-35	9.59*E*-33	59
Positive regulation of cell activation	79/670	421/18866	5.21*E*-35	1.33*E*-32	79
Regulation of cell-cell adhesion	80/670	439/18866	1.67*E*-34	4.05*E*-32	80
Regulation of lymphocyte proliferation	58/670	219/18866	2.19*E*-34	5.05*E*-32	58

**Table 5:** The information of the first 20 KEGG pathways enriched by downregulated CXCR2-relevant genes.

Description	GeneRatio	BgRatio	*p* value	FDR	Size
PPAR signaling pathway	2/9	76/8009	0.003064	0.085799	2
Nitrogen metabolism	1/9	17/8009	0.018951	0.191907	1
Vitamin digestion and absorption	1/9	24/8009	0.026662	0.191907	1
Aldosterone-regulated sodium reabsorption	1/9	37/8009	0.040838	0.191907	1
African trypanosomiasis	1/9	37/8009	0.040838	0.191907	1
Fat digestion and absorption	1/9	41/8009	0.045163	0.191907	1
Neuroactive ligand-receptor interaction	2/9	340/8009	0.053093	0.191907	2
Cholesterol metabolism	1/9	50/8009	0.054831	0.191907	1
Melanoma	1/9	72/8009	0.078097	0.24297	1
Protein digestion and absorption	1/9	95/8009	0.101876	0.251426	1
Pancreatic secretion	1/9	102/8009	0.109004	0.251426	1
Leukocyte transendothelial migration	1/9	112/8009	0.119099	0.251426	1
Systemic lupus erythematosus	1/9	133/8009	0.13997	0.251426	1
Cell adhesion molecules (CAMs)	1/9	147/8009	0.153638	0.251426	1
Breast cancer	1/9	147/8009	0.153638	0.251426	1
Gastric cancer	1/9	149/8009	0.155575	0.251426	1
Hepatitis C	1/9	155/8009	0.161361	0.251426	1
Tight junction	1/9	169/8009	0.174727	0.251426	1
Influenza A	1/9	170/8009	0.175674	0.251426	1
Alcoholism	1/9	184/8009	0.188836	0.251426	1

**Table 6:** The information of the first 20 biological processes enriched by downregulated CXCR2-relevant genes.

Description	GeneRatio	BgRatio	*p* value	FDR	Size
Forebrain regionalization	2/18	24/18866	0.000234	0.105833	2
Triglyceride catabolic process	2/18	38/18866	0.000592	0.105833	2
Regulation of gastrulation	2/18	43/18866	0.000759	0.105833	2
Neutral lipid catabolic process	2/18	48/18866	0.000945	0.105833	2
Acylglycerol catabolic process	2/18	48/18866	0.000945	0.105833	2
Regulation of sodium ion transmembrane transporter activity	2/18	55/18866	0.001239	0.110357	2
Regulation of sodium ion transmembrane transport	2/18	65/18866	0.001726	0.110357	2
Neural retina development	2/18	72/18866	0.002112	0.110357	2
Regulation of cardiac conduction	2/18	73/18866	0.002171	0.110357	2
Glycerolipid catabolic process	2/18	74/18866	0.00223	0.110357	2
Regulation of sodium ion transport	2/18	88/18866	0.003135	0.110357	2
Axis specification	2/18	88/18866	0.003135	0.110357	2
Regulation of neural precursor cell proliferation	2/18	91/18866	0.003348	0.110357	2
Dorsal/ventral pattern formation	2/18	92/18866	0.003421	0.110357	2
Triglyceride metabolic process	2/18	110/18866	0.004849	0.110357	2
Endocrine system development	2/18	125/18866	0.006216	0.110357	2
Camera-type eye morphogenesis	2/18	125/18866	0.006216	0.110357	2
Regulation of embryonic development	2/18	134/18866	0.007111	0.110357	2
Neutral lipid metabolic process	2/18	138/18866	0.007527	0.110357	2
Acylglycerol metabolic process	2/18	138/18866	0.007527	0.110357	2

## Data Availability

The data used to support the findings of this study are included within the supplementary information files.

## Abbreviations

TMB: Tumor mutation burden
CXCR: Chemokine receptor
TCGA: The Cancer Genome Atlas
GDC: Genomic Data Commons
GTEx: Genotype-Tissue Expression
GEPIA2: Gene Expression Profiling Interactive Analysis 2
OS: Overall survival
DSS: Disease-specific survival
MSI: Microsatellite instability
cBioPortal: cBio Cancer Genomics Portal
FDR: False discovery rate
GO: Gene Ontology
KEGG: Kyoto Encyclopedia of Genes and Genomes
TIMER: Tumor Immune Estimation Resource
GSEA: Gene set enrichment analysis
MsigDB: Molecular Signature Database
NES: Nominal enrichment score.

## Supplementary Materials

Supplementary Materials Supplementary Figure 1: prognostic implication of CXCR2 in diverse cancer types. (A) Kaplan-Meier curves of the OS differences between high and low expression of CXCR2 groups for ACC, DLBC, LAML, LGG, OV, and MESO. (B) Kaplan-Meier curves of the DSS differences between high and low expression of CXCR2 groups for ACC, LGG, OV, and STAD.
