# Supplementary figures and images for: Prognostic and Immunological Significance of CXCR2 in Ovarian Cancer: A Promising Target for Survival Outcome and Immunotherapeutic Response Assessment
Source: Dis Markers. 2021 Nov 19;2021:5350232. doi: 10.1155/2021/5350232 (PMC8626184; doi:10.1155/2021/5350232)

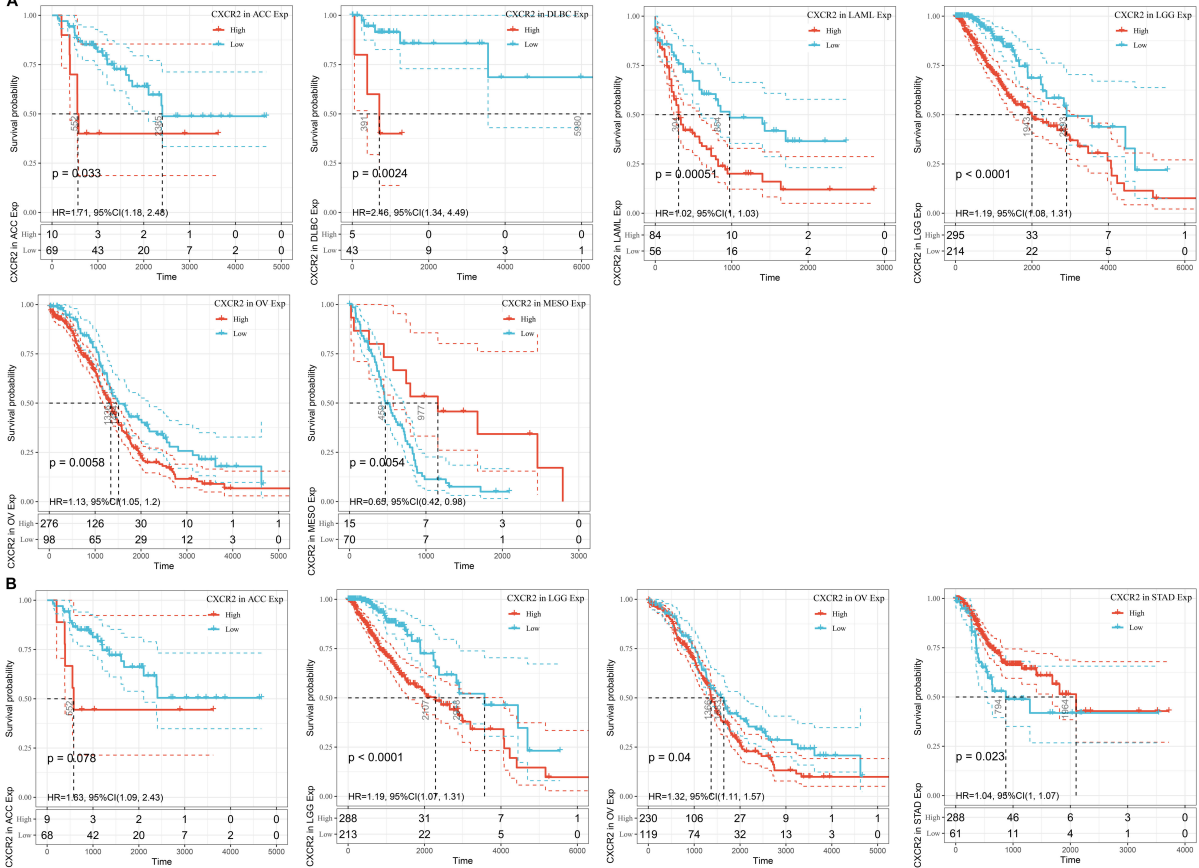

Supplement: Supplementary Materials — Supplementary Figure 1: prognostic implication of CXCR2 in diverse cancer types. (A) Kaplan-Meier curves of the OS differences between high and low expression of CXCR2 groups for ACC, DLBC, LAML, LGG, OV, and MESO. (B) Kaplan-Meier curves of the DSS differences between high and low expression of CXCR2 groups for ACC, LGG, OV, and STAD. [file 5350232.f1.pdf]
